# Cultural Considerations for the Adaptation of a Diabetes Self-Management Education Program in Cotonou, Benin: Lessons Learned from a Qualitative Study

**DOI:** 10.3390/ijerph18168376

**Published:** 2021-08-07

**Authors:** Halimatou Alaofè, Sarah Yeo, Abidemi Okechukwu, Priscilla Magrath, Waliou Amoussa Hounkpatin, John Ehiri, Cecilia Rosales

**Affiliations:** 1 Department of Health Promotion Sciences, Mel and Enid Zuckerman College of Public Health, The University of Arizona, Tucson, AZ 85724, USA; syeo@email.arizona.edu (S.Y.); aokechukwu@email.arizona.edu (A.O.); pmagrath@arizona.edu (P.M.); jehiri@email.arizona.edu (J.E.); 2 School of Nutrition and Food Science and Technology, Faculty of Agricultural Sciences, University of Abomey-Calavi (FSA-UAC), Campus d’Abomey-Calavi, Calavi 01 BP 526, Benin; amouswal@yahoo.fr; 3 Division of Public Health Practice & Translational Research, University of Arizona, Phoenix Plaza Building, 550 E. Van Buren Street, Phoenix, AZ 85006, USA; crosales@email.arizona.edu

**Keywords:** culturally tailored interventions, type 2 diabetes management, Meta Salud Diabetes, Cotonou, Benin

## Abstract

**Background**: Type 2 diabetes (T2D) poses a disproportionate burden on Benin, West Africa. However, no diabetes intervention has yet been developed for Benin’s contexts. This study aimed to explore specific cultural beliefs, attitudes, behaviors, and environmental factors to help adapt a diabetes self-management program to patients with T2D from Cotonou, in southern Benin. **Methods**: Qualitative data were collected through focus group discussions (FDGs) involving 32 patients with T2D, 16 academic partners, and 12 community partners. The FDGs were audio-recorded, transcribed verbatim from French to English, and then analyzed thematically with MAXQDA 2020. **Results**: Healthy food was challenging to obtain due to costs, seasonality, and distance from markets. Other issues discussed were fruits and vegetables as commodities for the poor, perceptions and stigmas surrounding the disease, and the financial burden of medical equipment and treatment. Information about local food selections and recipes as well as social support, particularly for physical activity, were identified, among other needs. When adapting the curriculum, gender dynamics and spirituality were suggested. **Conclusions**: The study demonstrates the need for culturally sensitive interventions and a motivation-based approach to health (spiritual and emotional support). It also lays the groundwork for addressing T2D contextually in Benin and similar sub-Saharan African countries.

## 1. Introduction

Type 2 diabetes (T2D) is now a global epidemic, with 15.9 million adults affected in sub-Saharan Africa (SSA), causing an annual economic cost of USD 3.3 billion. Furthermore, it is estimated that the burden of T2D on the African continent will increase by approximately 156% by 2045 [1]. Benin, a sub-Saharan country, is no exception as the prevalence of diabetes doubled between 2008 (4.6%) and 2015 (8.4%). In some regions, the diabetes prevalence reached 21.6%, with 15.1% in urban areas [2]. Moreover, the burden of T2D is projected to rise in Benin due to accelerated urbanization and a new lifestyle [3]. Indeed, diabetes ranks among the 10 most prevalent health conditions that cause disability, with a 55.8% rise in diabetes-related disabilities between 2007 and 2017 [4]. Unfortunately, most diabetes cases in Benin are in secondary health facilities where diabetes educators are scarce. Therefore, medical officers often assume these roles, while nurses also act as diabetes educators [5]. Consequently, T2D imposes a tremendous burden on individuals, their families, and healthcare systems in the country, indicating the need to identify interventions that provide optimal health care to persons living with T2D.

According to the Benin WHO STEPwise approach to surveillance survey (STEPS), 93.1% of the population consumed fewer than five servings of fruits and vegetables (F+V) daily, and only 29% participated in moderate-to-vigorous activity weekly [2]. High sodium intake, low whole-grain intake, and low F+V intake were the leading risk factors (38%) for death and disability-adjusted life years [6]. Moreover, the prevalence of hypertension (25.9%), hypercholesterolemia (18.2%), and overweight/obesity (23.2%) are alarmingly high in the country [3]. Regarding self-care behaviors, problems with adherence to diabetes treatment plans were encountered with diet (20%), physical activity (55.7%), and glycemic control (7.8%) [7], while only 9.1% of patients complied with the medication regimen [8]. Furthermore, a lack of integrated diabetes care teams created reactive instead of proactive approaches [9]. Thus, the care and management of diabetes should be patient-centered in light of the country’s context.

Despite Benin’s growing diabetes burden and needs, there are no system-wide interventions to aggressively address this problem or coordinated care aimed at better diabetes management [10,11]. Among the challenges are the training of health workers, organizational and structural features of the health system, and social and economic conditions. Currently, training models are based on a biomedical perspective, which neglects the psychosocial dimensions of illness [12]. In addition, many providers follow foreign practice guidelines imported from high-income countries without considering cultural differences, beliefs, perceptions, or resource limitations [13,14,15]. Many studies have stressed the importance of cultural awareness and sensitivity for successful health interventions in SSA [16,17]. Health in Benin is holistic, collective, and spiritual, while religion plays an important role in the lives of its citizens, with 28% Muslim, 25% Catholic, and 12% Vodun [9,18]. The use of religious-based and complementary/alternative health practices is widespread [19]. Therefore, an evidence-based diabetes management intervention that is culturally appropriate is needed to address the rising disease burden.

For this reason, we proposed to adapt Meta Salud Diabetes (MSD), an evidence-based diabetes self-management education program, to Benin’s context. MSD offers education on how to prevent disease complications through lasting behavioral changes [20]. Lifestyle changes and empowerment to control T2D are two important components of the program, both of which have been shown to improve blood sugar levels in SSA [21]. In addition, the program has effectively reduced diabetes risk factors in culturally diverse, low-income, and underserved Mexican-origin populations. The body mass index, waist circumference, LDL cholesterol, and glucose decreased significantly from baseline to three-month follow-up [22]. MSD also aligns with a systematic review that examines community ownership and mobilization as crucial factors for the sustainability of health interventions in SSA [13]. Finally, MSD addresses solution-focused and stress management approaches that were not studied in Benin but have proved effective elsewhere [23,24].

Scholars and practitioners consistently advise considering culture and context when developing interventions for minority populations with diabetes [16,17]. Thus, this study aimed to identify specific cultural beliefs, attitudes, behaviors, and environmental factors that could help inform a culturally and contextually appropriate program. In particular, this paper will offer specific recommendations on how MSD intervention components should be adapted culturally to improve participants’ knowledge, behavior, and health outcomes in Cotonou, Benin. To that end, we tried to understand the cultural factors that might influence the implementation of MSD based on the perspectives of three critical stakeholders: community and academic partners and diabetes patients.

## 2. Materials and Methods

### 2.1. Study Setting

This study was conducted between July and August 2019 in Cotonou, the economic capital of Benin, West Africa. The city is located in the Southeast of the country, between the Atlantic Ocean and Lake Nokoué. In Cotonou, French is the official language, and the population is estimated at 1.2 million [25]. Although the population grew by 12% from 2015 to 2018, most of the increase was among adults 45 years of age and older, which is significant since this age group is at a higher risk of T2D [25,26]. While the poverty rate for Benin was 49.5% (below USD 1.90 a day) in 2015, 98% of the population in Cotonou ranked in the last two quintiles of economic well-being [27]. Literacy rates for people between 15 and 49 years are at 67% (women) and 88.3% (men) [28]. Besides being Benin’s largest city, Cotonou is home to most of the government’s offices and diplomatic missions as well as small, private industries producing palm oil, beverages, and seafood processed at small scales. Cotonou is a city that has been rapidly changing, influencing lifestyles including diets with increased fats and sugar, reduced physical activity, and increased smoking, alcohol consumption, and stress [29]. As a result, between 2008 and 2015, the prevalence of diabetes has risen from 4.4% to 19% in Cotonou city [2].

### 2.2. Intervention Components

The MSD intervention consists of two-hour participatory workshop-style sessions delivered within a support group structure over 13 consecutive weeks and is described in detail in a previous publication [20]. During the weekly sessions, participants received educational information and participated in empowerment-building discussions and interactive workshop activities to promote long-term behavioral change related to disease complications, diet, and increased physical activity. Sessions maintained a basic structure throughout the 13 intervention weeks: blood pressure and glucose monitoring; readings, discussions, and games related to each week’s topic; execution of a custom-designed physical activity routine; and follow-up exercise to meet nutrition or physical activity goal. One or more health professionals (e.g., nurses, community health workers, doctors, or clinic staff, including interns) delivered each session during a regularly scheduled, face-to-face group meeting. The MSD program consists of seven components: (1) healthy eating (eat less fat, Healthy Plate to create a healthier diet); (2) being active (physical activity (30 min a day) adaptation); (3) monitoring blood sugar levels; (4) taking medication appropriately on a regular basis; (5) problem solving to maintain blood sugar levels in the targeted range; (6) reducing risks for diabetes-related complications (heart attack, stroke, kidney and nerve damage, vision loss); and (7) healthy-coping with stress. These components align with the Diabetes Self-Management Education and Support (DSMES) standards, as outlined in the 2016 American Diabetes Association (ADA) Standards of Medical Care in Diabetes [30,31].

### 2.3. Study Design

This qualitative study was conducted as part of a formative research effort to assess the contextual factors that might influence Benin’s MSD intervention delivery and inform necessary changes to the implementation strategy. In the present study, we conducted focus group discussions (FGDs) with T2D patients (>18 years of age), as well as community and academic partners to better understand the contextual factors influencing the implementation of the seven MSD components. The FGDs questions were derived from the literature and informed by the MSD curriculum (Supplementary Material 1, Questionnaire). In addition, the FGDs were conducted using a guide containing open-ended questions to allow the facilitators to ask questions to accommodate the discussion flow. Finally, the same questions were posed to all FDGs to allow for qualitative comparison among groups.

### 2.4. Participant Sampling

In order to participate in the study, participants had to live in Cotonou for at least six months each year. Their recruitment followed a previous quantitative survey, which collected data on the healthcare system’s capacity, socio-demographic and clinical characteristics, diabetes quality of life, and self-care behaviors among 300 T2D patients (age > 18 years) in four relevant secondary hospitals [32].

A purposive sampling strategy was used to recruit patients with T2D from the 300 patients who participated in the survey, with the aim of achieving demographic diversity in terms of sex, age, marital status, and education. Medical profiles of 300 patients with T2D were assessed for inclusion criteria, i.e., being diagnosed with T2D for more than a year, being 18 years and above, and willing to give informed consent to participate in the study. Exclusion criteria were patients with extreme disease conditions that would restrict them from participating in the study. After being assured of their congruousness with the inclusion criteria, 40 participants meeting the preliminary inclusion criteria were randomly selected and invited to participate in the study. For the health system’s capacity assessment, key informants were identified in collaboration with the Ministry of Health. During the process, community and academic partners were purposively selected for participation in this study to include partners of different cadres and experiences.

Study objectives were explained to the study participants, and those who agreed to participate gave their informed consent. A saturation of information was reached after three patient (9–11 participants per group), two community partner (6 participants per group), and two academic partner (8 participants per group) FGDs. In total, 12 community partners (10 community health workers (CHWs) and 2 traditional healers) and 16 academic partners (4 endocrinologists, 4 general physicians, 4 pharmacists, and 4 nutritionists) were identified and invited to participate. Of the 40 T2D patients formally invited by phone, 32 (80%) accepted the invitation. The FDGs were recorded with the consent of the interviewees and lasted roughly 2–2.5 h.

### 2.5. Training, Piloting, and Data Collectors

Two experienced facilitators and the lead author conducted the focus groups. Facilitators were secondary school teachers with bachelor’s degrees in education. The training involved a daylong instructional session and covered modules, including qualitative data collection methods and research ethics. The instruments used to collect data were tested in two ways. First, two CHWs and four Beninese nutrition experts reviewed the guide to ensure that the FGD questions were relevant, context specific, and culturally appropriate. Additionally, three pilot FGDs were conducted with four T2D patients, four CHWs, and four physicians to test the flow of questions among the target populations. Feedback was incorporated into the final version of the guides used for data collection. For example, feedback was given regarding the cultural relevance and translation accuracy of the questionnaire. Most respondents interpreted the questions differently than what was intended in the original questionnaire. Consequently, the questions were reworded. Our pretest trials also revealed the length of time needed to conduct the FGDs, which may have contributed to fatigue, disengagement, or distress. Academic partners and patients suggested keeping FGDs limited in time to participate after work. As a result, the sequence of administering the questionnaire was given attention, but most frequently, the approach remained routine in moving from informed consent to the demographic survey, followed by the main FGD scripts, which were simplified.

### 2.6. Data Collection Procedures

Every FGD began by introducing the MSD curriculum as a program designed to help individuals with T2D maintain their blood sugar levels within a healthy range [20]. The FDGs were conducted at a central location at the convenience of the participants. This location included a local primary school and a community-owned multipurpose building. In order to promote a more prosperous and open dialogue, the FGDs with patients were stratified by education level: one FGD involved primary-level patients and two involved secondary-level and higher education patients. FGDs were not stratified based on age, since the majority of patients were over 40 years of age. The community and academic partners were not stratified by gender, as it was deemed culturally acceptable for men and women of all ages to engage in the discussion. Facilitators reassured respondents that identifiable responses would not be disclosed. Additionally, they were trained on inclusive facilitation techniques to ensure all respondents’ active participation.

### 2.7. Data Analysis

FGDs were audio-recorded and transcribed verbatim from French into English. A bilingual French and English interpreter reviewed the transcripts for accuracy. Data analysis was initiated in the field, with debriefing meetings held following each FGD. With the help of the RAs, the lead author reviewed the collected data, identified new areas to explore further, and discussed topics that were saturated. Detailed field notes were taken on each FDG. Formal analysis was conducted at the end of data collection using content analysis [33] and was driven by deductive and inductive processes using MAXQDA 2020 (VERBI Software, Berlin, Germany, 2019) [34].

In the first step, a matrix of codes based on semi-structured FGD guides was developed, covering the seven components of MSD. Next, two independent researchers reviewed each transcript individually to check whether the codes were appropriate and identify recurring themes that were not part of the initial matrix. Finally, the researchers reviewed the codes produced by the process, agreed on the final codes, and grouped and categorized the codes accordingly. Researchers coded the first 50 lines of the scripts for all the FGDs using the coding system, assessing its relevance and consistency between researchers and determining whether the coding system required any modification. Disparities identified by the researchers were resolved through consensus, and more codes were added when deemed necessary. By the end of the process, all the scripts were coded using the finalized coding system. The codes were summarized by intervention components and participant groups. The findings were then compared between the three respondent groups [35]. The results of the study are reported according to the standards for qualitative research reporting (Supplementary Material 2) [36]. Demographic information was first entered on Microsoft Excel and exported to Stata, a quantitative analysis software (StataCorp, version 14), for tabulation.

## 3. Results

In total, seven FGDs with 60 participants were held (Table 1). Participants included T2D patients aged 44 and over (*n* = 3 FGs; 32 participants), community partners over 35 years old (*n* = 2 FGs; 12 participants), and academic partners (*n* = 2 FGs; 16 participants). The majority of participants in all three groups were married. The academic groups, however, were dominated by men, while women dominated the community partners. Finally, most academic partners worked for governments, whereas community and patient partners were mostly non-government employees.

Several sociocultural and environmental factors that may impact MSD intervention delivery were identified. The derived themes informed the cultural adaptation of each component of the MSD, as illustrated in Table 2.

### 3.1. Healthy Eating

Participants discussed multiple challenges to healthy eating, including access to healthy food, cultural norms and perceptions, and gender roles. All participants emphasized the relevance of information on locally available foods.

*Access to healthy food*. Participants mentioned cost, seasonality, and distance to healthy food markets as limitations to accessing them. These factors tend to be intertwined, as people need to travel farther to buy more affordable and healthy food.

“… *the decision about which fruit and vegetables to buy in the market is often based on the price, not the quality*.”

(Patient)

“*I have to go far from my house to buy cheaper fruits and vegetables*.”

(Patient)

“…. *because there are certain seasons when there are no fruits*.”

(Academic partner)

*Cultural perception and norms*. Along with the access to food, another recurring theme that was resonated among the participants was cultural perception and norms, especially concerning fruits and vegetables. Notwithstanding their nutritional benefits, vegetables and fruits have been considered commodities of the poor.

“*People think that eating vegetables is just being poor*.”

(Community partner)

“*Even though it is fruit season, people do not take it. Plenty go to the trash. For example, when you go to the north of the country, no one looks at mangoes*.”

(Community partner)

“*Well, because fruits and vegetables are not part of our culture. They are not food that can fill us up*.”

(Community partner)

*Gender role*. Specifically, among patient FDGs, the process and act of cooking was associated with gender, suggesting the importance of engaging women in the curriculum, especially for the healthy eating component.

“*Usually, our wives cook. My wife prepares for the house, goes to the market, and decides what to eat in the households*.”

(Patient)

*Lack of information on local foods and recipes*. The participants across the groups echoed the lack of culturally relevant information based on locally available food options.

“*Recipes based on our local foods will be helpful*.”

(Academic partner)

“*We need to focus on local diversity. Our local diversity is rich. You have to inform people. Not everything is expensive. People do not have the information of what is available*.”

(Academic partner)

### 3.2. Physical Activity and Exercise

The responses from the participants indicated that group support and neighborhood characteristics might facilitate engagement and participation. The respondents also voiced their preferences for easy and enjoyable activities to mitigate some of the patients’ challenges and guides for physical activities.

*Group support and neighborhood characteristics as a potential facilitator for engagement and participation*: Across all groups, many respondents commented that motivation and sustained participation in physical activities would mainly depend on within-group support. Specifically, the patient FGDs noted that neighborhood characteristics might determine participant engagement. Finally, many participants echoed that forming a group that functions as a sports club will improve engagement and participation in exercises and physical activities.

“*If we are all in the same geographic area, it will be easier. It is said that it is unnecessary to join a club to do sport. Why don’t we form this club*?”

(Patient)

“*The support among us is better than a family who does not have diabetes and does not know about the disease*.”

(Academic partner)

*Preferences for easy and enjoyable activities*. The majority of participants emphasized that they would prefer physical activities that are easy to follow due to the factors such as age, pre-existing conditions, and fatigue from work, and would prefer activities that are enjoyable to keep them motivated.

“*Easy activities because most diabetic patients are old or overweight*.”

(Community partner)

“*Not complicated activities because they complain that they are always tired*.”

(Academic partner)

“*Pick activities they will enjoy. Most adults need exercise to be fun, or they lose their motivation to do it over time*.”

(Community partner)

*Lack of guides for physical activities*: During the academic partner FGDs, participants noted that manual guides for physical exercise would provide examples of exercises for those who may not have adequate knowledge of which activities they could engage in.

“*If they can have some examples of physical activities, it will help a lot, specifically for those who do not have any activity*.”

(Academic partner)

### 3.3. Regular Blood Sugar Monitoring

Monitoring the blood sugar level seems to be often obstructed due to the limited availability of personal glucose monitoring test kits in the market, financial burden, and perspectives toward self-directed glucose monitoring.

*Availability of medical equipment in the market*. Participants, especially from the patient group, voiced their concern about the availability of medical equipment required to monitor blood sugar levels, such as strips and glucose meters.

“*I have five blood sugar meters. When I take one, there are no strips on the market, and I have to buy another*.”

(Patient)

“*There are different brands. A pharmacy sells glucometers, and they do not even have corresponding test strips*.”

(Academic partner)

*Financial burden*. One of the barriers to monitoring blood sugar levels that recurred among respondents was financial concerns. However, participants noted that support for the medical supplies could motivate participation in future programs and events.

“*I do not have a monitor, so I go to the hospital for my blood sugar when I have the money*.”

(Patient)

“*I will suggest not giving money directly but the devices, the glucometers that everyone needs to have at a lower cost*.”

(Patient)

“*I think that incentives such as free materials to use glucometers, blood tests, etc., can also motivate them*.”

(Academic partner)

*Perspectives toward self-directed glucose monitoring*. Participants also voiced that, in general, most people are not culturally accustomed to self-directed glucose monitoring and noted that this perspective might be a potential barrier to regular blood sugar monitoring.

“*They are afraid of sting/puncture, and it hurts*.”

(Community partner)

“*Glucometers, they do not use. They are afraid of blood*.”

(Community partner)

### 3.4. Adherence to Treatment Guidelines

The focus groups’ responses indicated that financial burden seems to be one of the most daunting challenges that impedes medication adherence. In addition, some participants expressed a lack of information on medications, while academic partners confirmed that they often do not have time during clinical consultations to explain and allay concerns about medications and other treatments for diabetic patients.

*Financial burden*. Participants discussed the financial burden and the significance of financial support for treatments and medical supplies to manage diabetes.

“*Some complaints about the burden of the cost of drugs*.”

(Community partner)

“… *there are drugs for treatment. For example, Diamicron costs 10,000 CFA and depending on the prescription, you can spend more than that a month. It will be great to have help with drugs*.”

(Patient)

“*Others are also afraid of going to the hospital because of the money. Therefore, they go to the traditional therapist*.”

(Community partner)

*Insufficient information during doctors’ consultation*. Doctors often find themselves without sufficient time to provide a detailed consultation, potentially leading to a lack of knowledge and awareness of adequate medication adherence.

“*Although doctors advise when it comes to medications, they do not have time to address some misconceptions about medications or explain their side effects*.”

(Academic partner)

“*I am told, if they inject insulin, it is not good for your body. Therefore, the doctor told me to never go on insulin and to continue with the drugs*.”

(Patient)

### 3.5. Problem Solving to Maintain Blood Sugar Levels in the Targeted Range

Patients with personal monitoring kits tend to manage blood sugar levels, quickly adjusting their diets accordingly. However, insufficient information on recommended diets and glycemic index of foods and poor portion control of foods, and difficulty making healthy choices when eating out seemed to be an impediment, particularly for those without the kits.

*Maintaining optimal blood sugar levels enabled by access to the monitoring kits*. Patients, especially those with the monitoring kits, were more likely to monitor blood sugar levels and adjust their diets regularly. They were able to set the goal for themselves to maintain sugar levels in the targeted range.

“… *I control my blood sugar almost every morning. When I have bad results, I try to see what went wrong yesterday in what I ate to find out where the problem is. I keep looking until I have the solution*.”

(Patient)

“*For example, my rate was 2.59 yesterday. Today I lowered it to 1.35. I reduced the amount of fruit and ate more of our vegetables, and mix with ‘tchairou’*.”

(Patient)

*Lack of information on nutrition values and glycemic index of foods*. Maintaining blood sugar levels in an optimal range could be challenging, as there was a lack of information on adequate diets recommended for T2D patients.

“*Due to the lack of information on food and cooking, I suffer from ulcers. Because of “do not eat this or that,” I do not know what to eat anymore. Sometimes I do not eat for four days, and I just drink water*.”

(Patient)

“*It looks like there are fruits rich in sugar, and some have low sugar levels. There is the information aspect, which we do not have*.”

(Patient)

*Challenges related to portion control of foods and making healthy choices when eating out*: Patients and academic partners reported the importance of considering local food options with the indication of portions when eating out in the context of Benin.

“*The environment is not favorable. At home, in terms of food, you can do whatever you can. However, when you come to town and go to the food vendor, you have to use what is available*.”

(Patients)

“*People with diabetes need reference bowls and portions suitable for Benin. Many eat two out of three meals outside the home*.”

(Academic partner)

### 3.6. Reducing Risks for Diabetes-Related Complications

Although respondents across the groups could provide their perceptions and experiences concerning the other themes such as foods, physical activities, and monitoring blood sugar levels, little was discussed and emerged concerning this component. Part of the reason could be attributed to a lack of awareness and knowledge, as hinted in the response below.

“*Diabetes affects many people in Cotonou. They die without knowing the reason. Most people rarely go to the hospital and many are sick all the time. Most of them have foot amputations. Also, people do not know what to do with the disease*.”

(Community Partner)

### 3.7. Coping with Stress

Negative perceptions toward diabetes, which may be a potential barrier to healthy coping with stress, were mentioned universally across the groups. The coping mechanisms that emerged included denial, religion, and focus on what is controllable.

*Negative perception toward the disease*. Of particular interest is the perception toward the disease, especially among the patient groups. Most of them perceived diabetes as a terminal disease with its complications as fatalistic. Diabetes was often associated with negative words such as the end of life and death, as stated below. Participants across the three focus groups mentioned that people who have diabetes might suffer additional social stigma issues. Participants had convergent thoughts that patients’ social stigma makes them reluctant to have open discussions about diabetes. It implies a greater perceived burden of the disease in the context, potentially leading to increased stress.

“*When they are told about diabetes, some parents think that we are already at the end of life and therefore that spending on drugs may be useless*.”

(Patient)

“*Even before the illness, I am told that it is the disease of the rich. These are the people who eat rich dishes, who drink champagne. That is why today, many people with diabetes do not like being known as such*.”

(Patient)

This statement resonated with other participants as well. Compared to other chronic diseases such as hypertension, participants reported a higher perceived severity for diabetes’ prognosis.

“*When you say you are hypertensive, it does not bother anyone. However, when you say you have diabetes, it is as if you will die tomorrow. You no longer have the support of the family*.”

(Patient)

*Denial*. Potentially due to the negative perceptions and social stigma associated with the disease, some patients seemed reluctant to accept the status, ignoring the fact that they have the disease.

“*I know it is a disease. However, to say that I have diabetes is like trying to give myself a name. I know I have diabetes, but I do not want to be told that I have diabetes*.”

(Patient)

“*The problem is that people do not want you to know they have diabetes. They think they are going to die anyway. Some do not care and do what they want*.”

(Community partner)

*Roles of religion*. When asked how they manage stress, religion seems to serve as a coping mechanism to drive away negative feelings or impulses.

“*I am happy that we are all Christians. Therefore, on the psychological side, we do not need help. Because if we are called Christians, we will not say that we are tired or want to kill ourselves or isolate ourselves from the world*.”

(Patient)

“*What is depression? For me, it is a matter of faith. There is a supreme being*.”

(Patient)

*Focusing on what is controllable*. Another strategy that participants utilize to cope with stress included focusing on what is controllable. For example, rather than focusing on negative feelings, patients were actively engaged in physical activities or monitoring the blood sugar level to distress during difficult periods.

“*What I do is to control my blood sugar almost every morning. When I have bad results, I tell myself that I have not done what I have to do. I run with friends*.”

(Patient)

## 4. Discussion

All too often, self-management interventions are not adequately matched to the intended target audience’s characteristics and circumstances in sub-Saharan Africa (SSA) [13,14,15]. Thus, tailoring MSD, a diabetes self-management education program, to meet the target population’s needs will likely increase usability, appeal, and effectiveness. As a result, this study will contribute to the development of a culturally sensitive program based on information specific to Benin. To the best of our knowledge, this is the first study to examine specific cultural beliefs, attitudes, behaviors, and environmental factors relevant to the adaptation of a self-directed diabetes intervention in Benin from the perspective of patients, academics, and community partners. We found broad consensus between the three groups. Patients, however, tended to give reasons for their behavior, whereas academic and community partners reported salient behaviors of patients. As Al Slamah et al. found in Saudi Arabia and Yeary et al. found in Marshall Island [37,38], sociocultural beliefs significantly influenced people with diabetes to accept their diagnosis and, thereby, participate in self-management interventions to improve disease outcomes.

Similar to the literature [39], this study reveals gaps between local perceptions and evidence-based nutritional research in healthy eating and access to healthy food. Cultural norms and gender roles influenced local perceptions. Specifically, despite their nutritional benefits, fruits and vegetables are not part of the dietary habits of the studied communities and are considered commodities of the poor. By reducing barriers such as lack of knowledge and skills to translate the recommendations into practice, a culturally appropriate diabetic meal plan will make it easier for people to adhere to the recommended diet. Consideration should also be given to environmental factors in Cotonou regarding healthy food, such as access, availability, and acceptability. Additionally, it will be necessary to consider crucial recommendations, such as choosing low-glycemic index foods and incorporating local foods. It will also be essential to include resources that facilitate adherence to the menus, such as recipes, cooking tips, a list of local foods, and where to obtain them, as suggested by Asaad et al. [40]. Finally, women were mentioned as having a significant role in food selection and preparation, indicating the importance of involving women in the intervention, as observed by Baig et al. [41].

All participants saw physical activity as critical to improving their diabetes outcomes. However, they noted that interventions to increase exercise would be more effective if patients received good social support, especially peer support and modules with enjoyable exercise routines. This finding is similar to Thomas et al.’s [42] conclusions that people with diabetes do not participate in physical exercise because of the difficulty of engaging in strenuous exercises and the distraction of other entertainment activities like watching television. In addition, busy schedules, inadequate structural facilities, and lack of time were perceived as barriers. The preference for physical activities in groups is in line with the Beninese cultural valuation of community-oriented activities [43]. Overall, interventions that promote physical activity should be flexible and adaptable to the environment of the participant group and must be enjoyable.

A patient’s ability to self-manage diabetes often hinges on adherence to treatment, good coping skills, and problem solving when their blood sugar targets are not being met. As previously observed in SSA [44,45], financial hardships impair patients’ ability to regularly monitor and maintain their blood sugar levels and adhere to medications and medical equipment. Therefore, a financial incentive could motivate them to maintain an optimal blood sugar level [46]. Specifically, access to personal monitoring kits can help patients regularly monitor and promptly adjust their diet based on the values they observe. However, isolated, vertical funding for diabetes alone may not be sustainable. A potential pathway to universal health coverage would protect beneficiaries, maximize financial protection, and prevent catastrophic health costs [47]. Despite the rising popularity of universal healthcare in policy discussions, less than one percent of Benin’s population is covered under these schemes [28]. A recent report from Kenya, Turkey, Mexico, Thailand, and China demonstrated early successes with the universal health coverage scale-up initiative [48]. Finally, yet importantly, participants used their cultural and religious beliefs to cope in stressful situations, suggesting that these beliefs could be used to improve coping and problem-solving skills.

This study also sheds light on negative social perceptions and stigmas associated with diabetes, which have significant implications, since they may lead to self-denial, hinder health-seeking behaviors, and negatively affect program acceptance. The findings indicate a need to sensitize the population and increase its awareness of the disease. Further attention should be given when publicly communicating the intervention to avoid unexpected adverse outcomes. Our study offers little evidence concerning reducing diabetes-related complications, reflecting a lack of awareness in the region regarding the specific complications and risk reduction [39]. As previously observed in SSA [49], many people with diabetes do not like to share their symptoms with other family members for fear of burdening them. Diabetes is often seen as a punishment by God and related to fatalistic beliefs. Thus, spiritual beliefs and discourses could be leveraged when delivering an intervention to encourage, motivate regular self-care, and reduce complications. Motivational support (both spiritual and emotional) is necessary, as psychological stress adversely affects health outcomes, mortality, and quality of life [50,51].

The study has several strengths and limitations. First, this study utilized qualitative comparison groups to elicit multiple perspectives involving patients, community partners, and academic partners [52]. Second, we were able to capture perspectives from diverse groups by using our unified interview guide. Third, in addition to demonstrating the need to adopt an evidence-based diabetes self-management curriculum to specific contexts, this study highlights the importance of considering contextual nuances. Fourth, we identified several barriers and facilitators that are common among different demographic groups. However, social desirability bias may have occurred if participants felt they were unable to express personal barriers. Another potential limitation is self-selection bias, because perhaps participants, by taking part in the study, were more open about sharing their experiences than those who did not; thus, the findings from the data may not represent the entire population. Additionally, we did not use a theoretical framework to direct our data collection and analysis. The FGDs questions were derived from the literature and informed by the MSD curriculum to understand the cultural factors that might influence the implementation of MSD. Finally, because this study was conducted in a specific region, its findings may not be generalized to other parts of the country.

### Implications for MSD Intervention

The findings of this study has implications for the success of the MSD program in Benin. Recommendations emerging from this study include the following:

- The MSD program must incorporate a meal menu plan that complies with the Benin nutrition therapy guidelines while taking into account factors such as access, acceptance, and accessibility of local foods. Additionally, the curriculum should include resources that facilitate adherence to the menu, such as recipes, cooking tips, a list of local foods and sources, and information on low-glycemic index foods.
- A revised curriculum could also gradually incorporate nutritious fruit snacks into the daily diet, introduce healthy foods patiently (e.g., vegetables), and ensure that portions are reasonable based on local bowls.
- Considering the DSME component of physical activity, findings suggest that Beninese physical activities should be based on group support and consist of easy and enjoyable activities like dancing to local music.
- Integrating glucose monitoring strategies within a collectivistic family framework will be essential for the MSD program to succeed. This strategy will involve the entire family in helping persons with diabetes monitor their blood sugar levels.
- Due to the lack of information regarding diabetes medications, clear guidance on how the medications work and are developed from traditional medicine will be imperative. Education on continuing medication will also be crucial, since Benin has a low compliance rate.
- Patients were reluctant to tell other family members about their diabetes symptoms for fear of burdening them. To combat these misconceptions, it will be crucial to stress the importance of being open with one’s family about diabetes symptoms for participants to ensure their long-term well-being. Early detection of symptoms and treatment can prevent significant problems and stressors in the long term.
- Patients with T2D also described the fatalistic belief that diabetes is God’s punishment. However, given the context of Benin, where religion plays a key role, spirituality and faith could be used to counter fatalistic beliefs.
- Given patients’ reticence to express their emotions, particularly outside of families, a MSD intervention must foster communication and trust (e.g., by being genuine with families and willing to help) to allow for discussions about stress and how to cope with it.
- Moreover, in addition to identifying specific cultural aspects of each DSME element, it will be essential to consider the matriarchal culture in Benin with distinct gender roles, since women are heavily involved in food preparation.

## 5. Conclusions

Ultimately, we can conclude that this study contributes in several ways to our understanding of the role of sociocultural norms, perceptions, and reality in implementing an evidence-based diabetes self-management education curriculum and provides a basis for adaptation to make it more effective. Additionally, this study fills a significant knowledge gap related to barriers and facilitators to the seven essential self-care behaviors in urban communities of Benin. Future intervention strategies should target the identified factors and emphasize the overlapping themes from our study and previous studies. Adding awareness of local foods and practices, adjusting cooking practices, and addressing stigma and gender roles associated with diabetes could be helpful when working with the surveyed communities. Additionally, as well as showing favorable views from key stakeholders about diabetes self-management programs, the findings highlight gaps in the healthcare system. We contend that this program could inform a more contextually specific self-management diabetes intervention, alleviate many of the challenges currently faced by diabetes care providers in Cotonou, and address the rising prevalence of T2D in the country.

## Figures and Tables

**Table 1:** Summary of respondent characteristics engaged in the focus groups, by respondent group.

Characteristics	T2D Patients (3 FGDs; *n* = 32)	Community Partners (2 FGDs; *n* = 12)	Academic Partners (2 FGDs; *n* = 16)
Age (years)			
Means	52.2	41.6	51.8
Range	44–59	36–44	41–62
Sex, *n* (%)			
Male	20 (62.5)	5 (41.7)	10 (62.5)
Female	12 (37.5)	7 (58.3)	6 (37.5)
Marital status, *n* (%)			
Single	4 (12.5)	5 (41.7)	2 (12.5)
Married	24 (75.0)	7 (58.3)	14 (87.5)
Separated/Divorced	2 (6.3)	0	0
Widowed	2 (6.3)	0	0
Occupation, *n* (%)			
Government employee	4 (12.5)	0	10 (62.6)
Non-government employee	14 (43.8)	8 (66.7)	3 (18.7)
Self-employed	5 (15.6)	2 (16.7)	3 (18.7)
Retired	5 (15.6)	0	0
Others ^a^	4 (12.5)	2 (16.7)	0

^a^ Student, housewife, unemployed.

**Table 2:** Summary of themes and issues emerging from the FGDs.

Component	Cultural Considerations
Healthy eating	Cost, seasonality, and distance to healthy food markets as barriers to accessing healthy food Fruits and vegetables are not part of culture and dietary habits. Fruits and vegetables are considered commodities of the poor. Women play key roles in food preparation, suggesting their engagement in the intervention beneficial. Lack of information on locally available food options
Physical activity and exercises	Group support and neighborhood characteristics as a potential facilitator for motivation and sustained participation Enjoyable and easy to follow physical activities due to age, pre-existing conditions, and fatigue from work Lack of manual guides to provide examples of exercises to follow
Regular blood sugar monitoring	Limited availability of medical equipment such as strips and glucose meters Financial burden People are not culturally accustomed to self-directed glucose monitoring.
Adherence to treatment guidelines	Financial burden Lack of information on medications Physicians do not have time to explain and discuss concerns about medications and other treatments for diabetic patients.
Problem solving	Lack of information on adequate diets recommended for T2D patients Challenges related to portion control of foods and making healthy choices when eating out
Reducing risks for diabetes-related complications	Little discussion on the complications due to lack of awareness and knowledge
Coping with stress	Perceive diabetes and its complications as a terminal disease leading to fatalistic views Patients’ social stigma made them reluctant to have open discussions about diabetes. Reluctant to accept the status Religion serves as a coping mechanism to drive away negative feelings or impulses. Focusing on what is controllable

## Data Availability

The datasets used and/or analyzed during the current study are available from the corresponding author on reasonable request.

## Supplementary Materials

The following are available online at https://www.mdpi.com/article/10.3390/ijerph18168376/s1, Supplementary Material 1: Questionnaire. Meta Salud Diabetes focus group guide; Supplementary Material 2: Checklist for Standards for Reporting Qualitative Research (SRQR) for research study on the cultural adaption of the Meta Salud Diabetes program.

## References

[1] Kibirige D., Lumu W., Jones A.G., Smeeth L., Hattersley A.T., Nyirenda M.J. (2009). Understanding the manifestation of diabetes in sub Saharan Africa to inform therapeutic approaches and preventive strategies: A narrative review. Clin. Diabetes Endocrinol..

[2] WHO (2015). WHO STEPwise Approach to Surveillance (STEPS).

[3] Delisle H., Ntandou-Bouzitou G., Agueh V., Sodjinou R., Fayomi B. (2012). Urbanisation, nutrition transition and cardiometabolic risk: The Benin study. Br. J. Nutr..

[4] Institute for Health Metrics and Evaluation (IHME) (2017). Benin. http://www.healthdata.org/benin.

[5] Adeya G., Bigirimana A., Cavanaugh K., Franco L.M. (2007). Rapid Assessment of the Health System in Benin.

[6] GBD Risk Factors Collaborators (2020). Global burden of 87 risk factors in 204 countries and territories, 1990–2019: A systematic analysis for the Global Burden of Disease Study 2019. Lancet.

[7] Alassani A., Dovonou C., Gninkoun J., Wanvoegbe A., Attinsounon C., Codjo L., Zannou M., Djrolo F., Houngbe F. (2017). Perceptions and practices of people with diabetes mellitus at the Centre National University Hospital Hubert Maga Koutoucou Cotonou. Le Mali Med..

[8] Wanvoegbe F.A., Agbodande K.A., Alassani A., Aviansou A., Gninkoun J., Amoussou-Guenou D., Djrolo F., Zannou M., Houngbe F. (2018). Evaluation de l’observance thérapeutique chez les diabétiques au Bénin. Médecine D’afrique Noire.

[9] Scott A., Ejikeme C.S., Clottey E.N., Thomas J.G. (2013). Obesity in sub-Saharan Africa: Development of an ecological theoretical framework. Health Promot. Int..

[10] Owolabi M., Miranda J.J., Yaria J., Ovbiagele B. (2016). Controlling cardiovascular diseases in low and middle income countries by placing proof in pragmatism. BMJ Glob. Health.

[11] Ebireri J., Aderemi A.V., Omoregbe N., Adeloye D. (2016). Interventions addressing risk factors of ischaemic heart disease in sub-Saharan Africa: A systematic review. BMJ Open.

[12] Cappuccio F.P., Miller M.A. (2016). Cardiovascular disease and hypertension in sub-Saharan Africa: Burden, risk and interventions. Intern. Emerg. Med..

[13] Iwelunmor J., Blackstone S., Veira D., Nwaozuru U., Airhihenbuwa C., Munodawafa D., Kalipeni E., Jutal A., Shelley D., Ogedegbe G. (2016). Toward the sustainability of health interventions implemented in sub-Saharan Africa: A systematic review and conceptual framework. Implement. Sci..

[14] Miranda J.J., Zaman M.J. (2010). Exporting ‘failure’: Why research from rich countries may not benefit the developing world. Rev. Saude Publica.

[15] O’Donnell O. (2007). Access to health care in developing countries: Breaking down demand side barriers. Cad. Saúde Pública.

[16] Rawal L.B., Tapp R.J., Williams E.D., Chan C., Yasin S., Oldenburg B. (2012). Prevention of type 2 diabetes and its complications in developing countries: A review. Int. J. Behav. Med..

[17] Mathews E., Thomas E., Absetz P., D’Esposito F., Aziz Z., Balachandran S., Daivadanam M., Thankappan K.R., Oldenburg B. (2018). Cultural adaptation of a peer-led lifestyle intervention program for diabetes prevention in India: The Kerala diabetes prevention program (K-DPP). BMC Public Health.

[18] The World Factbook—Central Intelligence Agency. www.cia.gov.

[19] Kpozehouen A., Djrolo F., Sossa C.J., Gbary A.R., Chouehanou Y., Fambo D., Tchabi Y., Salamon R., Houinato D.S. (2015). Prevalence and Associated Factors of Diabetes Mellitus in Benin. Open J. Epidemiol..

[20] Sabo S., Champion C.D., Bell M.L., Vucovich E.C., Ingram M., Valenica C., Vasquez M.D.C.C., Gonzalez-Fagoaga E., De Zapien J.G., Rosales C.B. (2018). Meta Salud Diabetes study protocol: A cluster-randomised trial to reduce cardiovascular risk among a diabetic population of Mexico. BMJ Open.

[21] Mogueo A., Oga-Omenka C., Hatem M., Kuate Defo B. (2020). Effectiveness of interventions based on patient empowerment in the control of type 2 diabetes in sub-Saharan Africa: A review of randomized controlled trials. Endocrinol. Diabetes Metab..

[22] Denman C.A., Rosales C., Cornejo E., Bell M.L., Munguía D., Zepeda T., Carvajal S., de Zapien J.G. (2014). Evaluation of the community-based chronic disease prevention program Meta Salud in Northern Mexico, 2011–2012. Prev. Chronic Dis..

[23] Viner R.M., Christie D., Taylor V., Hey S. (2003). Motivational/solution-focused intervention improves HbA1c in adolescents with Type 1 diabetes: A pilot study. Diabet. Med..

[24] Zamani-Alavijeh F., Araban M., Koohestani H.R., Karimy M. (2018). The effectiveness of stress management training on blood glucose control in patients with type 2 diabetes. Diabetol. Metab. Syndr..

[25] Ministère de la santé du Bénin (2019). Annuaire des Statistiques Sanitaires 2018.

[26] Ministère de la santé du Bénin (2017). Annuaire des Statistiques Sanitaires 2016.

[27] Ministère de la santé du Bénin (2018). Strategie Nationale D’action sur les Determinants Sociaux de la Sante Cotonou.

[28] Institut National de la Statistique et de l’Analyse Économique (INSAE) and ICF (2019). République Du Bénin Ciquième Enquête Démographique et de Santé au Bénin (EDSB-V) 2017–2018.

[29] Affogbolo I., Ologoudou E. (2004). Cotonou: Regards Sur Une Ville.

[30] Funnell M.M., Brown T.L., Childs B.P., Haas L.B., Hosey G.M., Jensen B., Maryniuk M., Peyrot M., Piette J.D., Reader D. (2008). National standards for diabetes self-management education. Diabetes Care.

[31] Prevention I. (2016). Diabetes Association Standards of Medical Care in Diabetes. Diabetes Care.

[32] Alaofè H., Hounkpatin W.A., Djrolo F., Ehiri J., Rosales C. (2021). Knowledge, attitude, practice and associated factors among patients with type 2 diabetes in Cotonou, Southern Benin. BMC Public Health.

[33] Elo S., Kyngäs H. (2008). The qualitative content analysis process. J. Adv. Nurs..

[34] VERBI Software (2019). MAXQDA 2020 (Computer Software).

[35] Lindsay S. (2019). Five Approaches to Qualitative Comparison Groups in Health Research: A Scoping Review. Qual. Health Res..

[36] O’Brien B.C., Harris I.B., Beckman T.J., Reed D.A., Cook D.A. (2014). Standards for reporting qualitative research: A synthesis of recommendations. Acad. Med..

[37] Al-Slamah T., Nicholl B.I., Alslail F.Y., Harris L., Melville C.A., Kinnear D. (2020). Cultural adaptation of self-management of type 2 diabetes in Saudi Arabia (qualitative study). PLoS ONE.

[38] Yeary K.H.C.K., Aitaoto N., Sparks K., Ritok-Lakien M., Hudson J.S., Goulden P., Bing W., Riklon S., Rubon-Chutaro J., Mcelfish P.A. (2017). Cultural Adaptation of Diabetes Self-Management Education for Marshallese Residing in the United States: Lessons Learned in Curriculum Development. Prog. Community Health Partnersh. Res. Educ. Action.

[39] Stephani V., Opoku D., Beran D. (2018). Self-management of diabetes in Sub-Saharan Africa: A systematic review. BMC Public Health.

[40] Asaad G., Soria-Contreras D.C., Bell R.C., Chan C.B. (2016). Effectiveness of a Lifestyle Intervention in Patients with Type 2 Diabetes: The Physical Activity and Nutrition for Diabetes in Alberta (PANDA) Trial. Healthcare.

[41] Baig A.A., Benitez A., Quinn M.T., Burnet D.L. (2015). Family interventions to improve diabetes outcomes for adults. Ann. N. Y. Acad. Sci..

[42] Thomas N., Alder E., Leese G.P. (2004). Barriers to physical activity in patients with diabetes. Postgrad. Med. J..

[43] Balint G.P., Buchanan W.W., Dequeker J. (2006). A brief history of medical taxonomy and diagnosis. Clin. Rheumatol..

[44] Hall V., Thomsen R., Henriksen O., Lohse N. (2011). Diabetes in Sub Saharan Africa 1999-2011: Epidemiology and public health implications. A systematic review. BMC Public Health.

[45] Adu M.D., Malabu U.H., Malau-Aduli A.E.O., Malau-Aduli B.S. (2019). Enablers and barriers to effective diabetes self-management: A multi-national investigation. PLoS ONE.

[46] Kalra S., Jena B.N., Yeravdekar R. (2018). Emotional and Psychological Needs of People with Diabetes. Indian J. Endocrinol. Metab..

[47] World Health Organization (WHO) (2010). Health Systems Financing: The Path to Universal Coverage.

[48] Jan S., Laba T.L., Essue B.M., Gheorghe A., Muhunthan J., Engelgau M., Mahal A., Griffiths U., McIntyre D., Meng Q. (2018). Action to address the household economic burden of non-communicable diseases. Lancet Lond. Engl..

[49] Hushie M. (2019). Exploring the barriers and facilitators of dietary self-care for type 2 diabetes: A qualitative study in Ghana. Health Promot. Perspect..

[50] Colchamiro R., Ghiringhelli K., Hause J. (2010). Touch Hearts, Touching Minds: Using Emotion-based Messaging to Promote Healthful Behavior in the Massachusetts WIC Program. J. Nutr. Educ. Behav..

[51] Lustman P.J., Anderson R.J., Freedland K.E., DeGroot M., Carney R.M., Glouse R.E. (2000). Depression and Poor Glycemic Control: A Meta-Analytic Review of the Literature. Diabetes Care.

[52] Carter N., Bryant-Lukosius D., Dicenso A., Blythe J., Neville A.J. (2014). The use of triangulation in qualitative research. Oncol. Nurs. Forum.

