# Supplementary material for: Cultural Considerations for the Adaptation of a Diabetes Self-Management Education Program in Cotonou, Benin: Lessons Learned from a Qualitative Study
Source: Int J Environ Res Public Health. 2021 Aug 7;18(16):8376. doi: 10.3390/ijerph18168376 (PMC8393923; doi:10.3390/ijerph18168376)
Supplement: Supplementary file 1 [file ijerph-18-08376-s001.zip › Supplementary Material 1. Questionnaire Meta Salud Diabetes Focus Group Guide.pdf]

Questionnaire: Meta Salud Diabetes Focus Group Guide

The Meta Salud Diabetes (MSD) intervention is based on seven main components:

1. **Healthy eating**:
   - The MSD recommends eating fruit and vegetables. Do you think you will be able to add fruits and vegetables to your diet? Why do you think it will be difficult or easy to increase your consumption of fruits and vegetables? What are the important elements that affect your consumption of fruits and vegetables (resources, purchasing capacity/income, family composition, etc.)?
   - The program also proposed to use "Healthy Plate" to better control your diabetes. Do you think you can use the method regularly? If not applied, what other method can be efficient?
   - How to approach the problem of nutrition for people with diabetes in Cotonou? Do you think that cooking classes can help? What about cooking demonstrations? Or sitting with a dietician/nutritionist for dietary advice?

2. **Physical activity**: What do you think of MSD physical activities? What kind of physical activity will you prefer? Will it be easy for you to participate in physical activities with your families? Or will you prefer to go to clubs and have a trainer? What can motivate you to do more exercise?

3. **Regular Blood Sugar Monitoring**: Why most diabetic patients did not monitor their blood sugar? How can we motivate you to regularly monitor your blood sugar to control your diabetes?

4. **Adherence to Treatment Guidelines**: Why most diabetic patients did not buy/consume their medication? How can we motivate you to buy your medications and follow the doctor's recommendations regularly? Why do most patients use traditional/local medicine instead of "Western medication”?

5. **Problem Solving**: How do you find solutions to solve the problems you are going through because of diabetes?

6. **Reducing risks for diabetes-related complications**: We all know that if we do not control our diabetes, we can suffer different complications such as heart disease, imputations. How do you manage to reduce the risk of these diseases?

7. **Coping with stress**: How do you manage stress caused by the burden of their diabetes?
